# Sulfone-decorated hypercrosslinked polymers for sacrificial light-driven hydrogen evolution from water

**DOI:** 10.1039/d6ta01673a

**Published:** 2026-06-26

**Authors:** Paul Schweng, Dominik Eder, Reiner Sebastian Sprick, Alexey Cherevan, Robert T. Woodward

**Affiliations:** a Institute of Materials Chemistry and Research, Faculty of Chemistry, University of Vienna Währinger Straße 42 1090 Vienna Austria robert.woodward@univie.ac.at; b Vienna Doctoral School in Chemistry, University of Vienna Währinger Straße 42 1090 Vienna Austria; c Institute of Materials Chemistry, TU Wien Getreidemarkt 9/BC 1060 Vienna Austria alexey.cherevan@tuwien.ac.at; d Department of Pure and Applied Chemistry, University of Strathclyde Glasgow G1 1XL Scotland UK sebastian.sprick@strath.ac.uk

## Abstract

Polymer photocatalysts have emerged as a versatile class of materials for solar-driven hydrogen evolution, offering tunable optoelectronic properties, structural diversity, and synthetic flexibility. Unlike inorganic semiconductors, polymer photocatalysts can be molecularly engineered to optimise light absorption, charge separation, and energy levels as well as porosity through precise control of their composition and architecture. One of the challenges of this material class is the fact that highly functionalised monomers are used, for example, multifunctional bromo and boronic acid compounds, which are then coupled using Suzuki–Miyaura polycondensation reactions that require Pd(0) catalysts. Similarly, highly active covalent organic frameworks require highly functionalised building blocks which are made using costly multi-step synthesis. Herein, we overcome this limitation of photoactive polymers by preparing swellable hypercrosslinked polymers (HCPs) that combine photocatalytic activity with high accessible surface areas, which are made using inexpensive monomers and not relying on noble metals for their synthesis. The HCPs contain dibenzo[*b*,*d*]thiophene sulfone, which makes the materials photoactive, and we find that the material that is made in a 2 : 1 ratio of 4,4′-bis(chloromethyl)-1,1′-biphenyl and dibenzo[*b*,*d*]thiophene sulfone gives the highest photocatalytic activity of 249 ± 41 µmol h^−1^ g^−1^. Although materials appear unstable under photocatalytic conditions, we are able to overcome this through the addition of a radical scavenger, which results in increased stability of the system but also increases the activity of the system to 275 ± 58 µmol h^−1^ g^−1^. This study showcases the potential of this inexpensive and readily available material class for photocatalytic hydrogen evolution from water.

## Abstract 2

Sulfone-containing hypercrosslinked polymers are shown to be a versatile class of materials for solar-driven hydrogen evolution, coupling structural diversity with simple synthesis.

## Graphical abstract

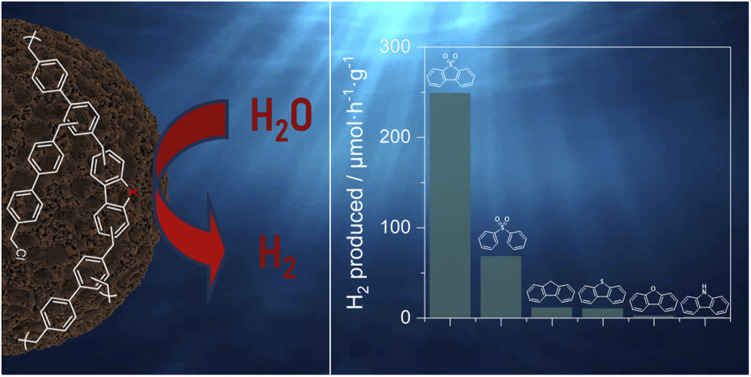

## Introduction

The production of hydrogen from water remains an area of immense interest due to the need for sustainable and storable fuels for the energy transition. Hydrogen can be used in fuel cells to generate electricity when needed by power grids or directly in propulsion. Importantly, the production of hydrogen fuel needs to avoid the production of greenhouse gases; however, currently the majority of hydrogen is produced using processes that use large amounts of energy and also produce carbon dioxide directly, *i.e.* the water–gas shift reaction and methane steam reforming.^1^ A potential approach to overcome these issues is the use of photocatalytic water splitting. Here, a photocatalyst uses water and light to drive hydrogen production without the emission of greenhouse gases in the process.^2^

Much of the research in this area has been dedicated towards the development of inorganic semiconductor materials,^3^ and has also led to the development of scalable panels, which have been used on 100 m^2^-scale.^4^ Despite the progress in this area, efficiencies in terms of their solar-to-hydrogen efficiency remain low (>1%), primarily because many of these highly developed systems only absorb UV light,^2^ which makes up less than 5% of the solar spectrum at sea level.^5^

Costs arising from the material production and in particular the highly optimised co-catalysts systems are potentially very high, which has resulted in the search for alternatives such as photocatalytically active natural minerals.^6^ Considering synthetic approaches, organic semiconductors have emerged as competitive photocatalysts for hydrogen evolution from water over the last decade.^7–10^ Interest in these materials originates from their potential structural diversity, which allows for controlled tuning of the materials' opto-electronic properties. This has resulted in the exploration of a wide range of organic photocatalysts for hydrogen evolution from water, including carbon nitrides,^11,12^ unbranched conjugated polymers,^13^ small molecules/oligomers,^14–16^ conjugated microporous polymers (CMPs),^17,18^ covalent triazine-based frameworks (CTFs),^19,20^ and covalent organic frameworks (COFs).^21,22^ Unbranched conjugated polymer photocatalysts can also be made solution-processible through the introduction of solubilising side-chains. This has been exploited in making complex materials that can be used as photoactive films as well as in making bulk heterojunction photocatalysts (BHJ) consisting of small molecules and conjugated polymers.^23,24^ BHJ photocatalysts show remarkable efficiencies for sacrificial hydrogen production from water, with many of the most efficient polymer photocatalysts falling into this category.

For future scale up and the transition from the laboratory-to-fabrication it will be important to reduce the carbon footprint and the embedded carbon in these conjugated materials.^25^ The use of multi-step synthesis also makes these materials potentially economically uncompetitive due to their high production costs.^26^ The use of palladium in the synthesis of polymer photocatalysts, when using Suzuki–Miyaura, Sonogashira, or Heck-coupling reactions, is also a considerable factor in the environmental impact as well as costs when making these materials – in particular once scaled up.^27^ CTFs and COFs can be made using condensation reactions that do not require palladium, producing materials with high activities for photocatalytic hydrogen production from water.^28^ However, their production can demand relatively harsh conditions and multi-step synthesis of monomers is often required, which effects their cost and carbon impact negatively.

Hypercrosslinked polymers (HCPs) have been reported as materials for sorption and separation processes,^29^ catalysis,^30^ and water capture from air.^31,32^ These materials are obtained through crosslinking of simple building blocks often using Friedel–Crafts reaction, which results in materials with high surface areas, which makes these competitive but much cheaper alternatives to other organic polymeric alternatives.^33^ The synthesis of HCPs is highly modular, allowing for the tuning of material properties through co-polymerisation and reduced costs as building blocks do not have to be highly functionalised to be polymerised.

Given that small molecules and oligomers have shown potential for the production of solar fuels^14–16^ it is not surprising that the crosslinking of small molecules has been reported to give photocatalytically active materials for CO_2_ photoreduction,^34,35^ hydrogen peroxide production,^36,37^ dye degradation,^38^ and organic transformations.^39,40^ The use of HCPs in photocatalysis is also of interest as these materials have shown to undergo swelling in solvent.^41,42^ In the context of photocatalysis, swelling has been shown to increase the interfacial surface area which results in materials with increased activity for photocatalytic hydrogen production^43^ if it can be achieved using aqueous solutions used in photocatalysis.

Here, we report the use of HCPs for sacrificial hydrogen production from water using dibenzo[*b*,*d*]thiophene sulfone as a building block due to the large number of reports that show high activity of the resulting photocatalysts, including unbranched polymers,^44,45^ conjugated microporous polymers,^46,47^ CTFs^48^ and COFs.^49,50^ By systematically varying the linker-to-monomer ratio, we investigate how composition influences textural properties, optoelectronic behaviour, photocatalytic activity, and stability, thereby establishing structure–performance relationships for this inexpensive and synthetically accessible class of materials.

## Results and discussion

**Fig. 1:**
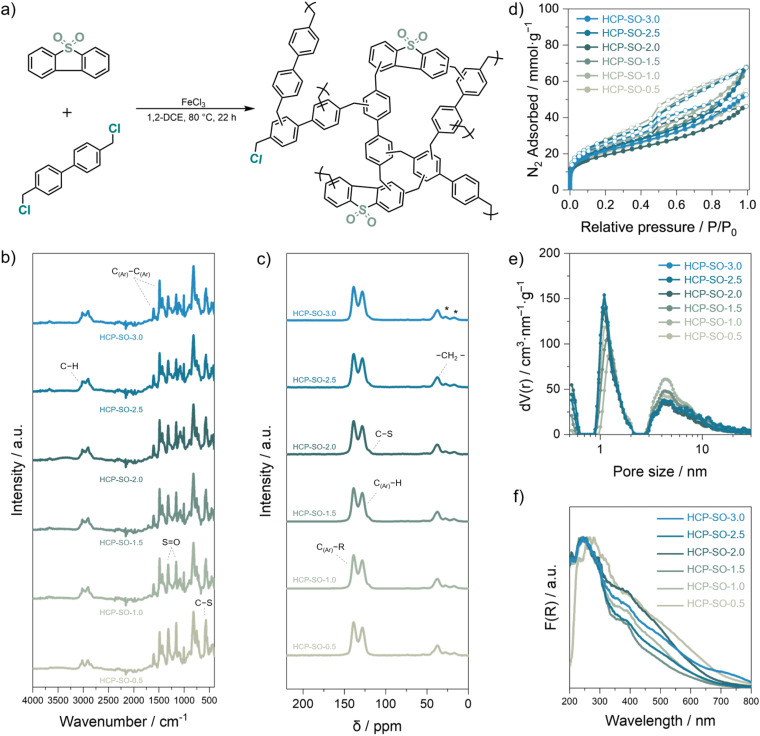
(a) Reaction scheme for the synthesis of the HCP-SOs photocatalysts. Note that the structure of the product is not necessarily representative, (b) FT-IR spectra of HCP-SOs, (c) ^13^C CP/MAS ssNMR spectra of HCP-SOs. * Indicates spinning sidebands, (d) N_2_ sorption isotherms of HCP-SOs measured at −196 °C. Filled circles indicate adsorption and empty circles indicate desorption. (e) QSDFT pore size distribution, and (f) UV/vis diffuse reflectance spectra of HCP-SOs.

A series of six hypercrosslinked polymers containing the dibenzo[*b*,*d*]thiophene sulfone motif was synthesised by co-polymerising with varied amounts of 4,4′-bis(chloromethyl)-1,1′-biphenyl acting as the crosslinking agent in the presence of FeCl_3_ (Fig. 1a). The copolymers allowed us to investigate the effect of composition on textural and chemical properties, and performance as a photocatalyst for hydrogen evolution from water. The resulting networks were thoroughly washed with methanol to remove unreacted monomers, small oligomers, and coupling reagents. This was followed by drying under reduced pressure at 80 °C to yield HCP-SO-*X*.*Y*, where *X*.*Y* denotes the nominal linker/monomer ratio, *i.e.* the millimolar amount of 4,4′-bis(chloromethyl)-1,1′-biphenyl relative to dibenzo[*b*,*d*]thiophene sulfone which was kept constant at 1 mmol. Further synthesis details are provided in the Experimental Section 2 of the SI.

Fourier-transform infrared spectroscopy (FT-IR, Fig. 1b) was used to characterise the materials. Characteristic peaks for methylene bridges formed during the condensation reaction were identified at 3020 cm^−1^ and 1463 cm^−1^ corresponding to C–H stretching and bending vibrations, respectively. Peaks at 1318 cm^−1^, 1158 cm^−1^, and 568 cm^−1^ were assigned to S=O and C–S stretching vibrations, strongly indicating the presence of sulfone groups in the materials. ^13^C cross-polarisation/magic angle spinning solid-state NMR (CP/MAS ssNMR) also agrees with the formation of an HCP network material (Fig. 1c). A signal at ∼38 ppm was assigned to methylene bridges, and signals at ∼129 and ∼138 ppm to aromatic (C_Ar_–H) and substituted aromatic carbons (C_Ar_–R), respectively. The signal at ∼120 ppm (in the shoulder of a larger peak) was attributed to the C–S bond of the dibenzo[*b*,*d*]thiophene sulfone moiety. Neither significant differences in functional groups nor structure within the series could be discerned from the FT-IR and ssNMR spectra.

Further analysis of the chemical composition of the HCP-SOs was undertaken by performing X-ray photoelectron spectroscopy (XPS, Fig. S2). The primary feature in the high-resolution C 1s spectrum at a binding energy of 284.8 eV was attributed to C–C bonding, encompassing both sp^3^ and sp^2^ carbon. A less prominent peak is evident at 286.3 eV, indicative of C–S bonds, while a broad, low-intensity π–π* shake-up feature emerged at 291.1 eV in all cases. The high-resolution S 2p spectrum revealed asymmetrically shaped peaks at binding energies of 168.3 eV and 169.4 eV assigned to S 2p_3/2_ and S 2p_1/2_, respectively, characteristic for sulfone moieties.

XPS and elemental microanalysis (EA) data was used to determine the surface and bulk chemical composition of HCP-SOs (Tables S2 and S3). Increasing the linker/monomer ratio resulted in a reduced sulfur content in EA, with HCP-SO-0.5 exhibiting the highest concentration at 1.87 ± 0.07 wt%, while HCP-SO-3.0 had the lowest at 0.98 ± 0.03 wt%. A similar trend was observed in the XPS data, which revealed slightly higher overall sulfur content but still lower than the expected values assuming complete conversion (Table S4). The incorporation efficiency of dibenzo[*b*,*d*]thiophene sulfone was below 30% for all samples. In contrast, the experimentally determined crosslinking density remained essentially constant across the series, despite the expected variation in nominal values (Table S5). Further discrepancies between EA and XPS can potentially be ascribed to residual water adsorption prior to EA analysis, and the contribution of adventitious carbon to XPS signals.

Nitrogen sorption isotherms were measured at −196 °C to probe the textural properties of HCP-SOs (Fig. 1d). All polymers displayed characteristics of both type I and type IVa isotherms, indicative of micro- and mesoporosity, as well as H2-type hysteresis, characteristic for networks with broad pore size distributions and narrow pore necks. The Brunauer–Emmett–Teller (SA_BET_) specific surface areas (Table S6) showed minimal variation across the series, with HCP-SO-0.5 displaying the lowest surface area at 1511 ± 49 m^2^ g^−1^ and HCP-SO-2.5 the highest at 1681 ± 6 m^2^ g^−1^. Quenched solid density functional theory (QSDFT) analysis (Fig. 1e) showed a multimodal pore size distribution across all networks, with the majority of pores concentrated in the micropore region of around 1 nm. Notably, a shift towards smaller pore sizes was observed with higher linker ratios, marked by a noticeable decrease in mesoporosity and an increase in microporosity. Thermogravimetric analysis (TGA) under N_2_ atmosphere was performed to assess the thermal stability of HCP-SOs (Fig. S3). Negligible weight loss was observed until ∼300 °C in all cases and no significant variations are noticeable across the series.^51^ All materials were found to be amorphous as evident from their featureless powder X-ray diffraction patterns (Fig. S4). Dynamic light scattering measurements of HCP-SO-2.0 revealed a broad particle size distribution centred around 900 nm (Fig. S5).

We measured their opto-electronic properties using UV-vis diffuse reflectance spectroscopy (DRS, Fig. 1f) and photoluminescence spectroscopy (PL, Fig. S6). In all cases, DRS spectra show absorption over the entire UV-vis range, reaching as far as 800 nm. Apart from the main absorption maximum centred in the 250–300 nm range, all polymers feature multiple absorption shoulders with the most prominent transitions around 360–390 nm. We also calculated the apparent optical band gaps of the HCP-SOs from their DRS spectra using Tauc plots (Fig. S7). The derived values ranged from 1.24 to 1.46 eV, which lies within the desirable range for water splitting reactions. We note, however, that these values should be regarded as estimates given the amorphous nature and the inherent structural inhomogeneity of the samples. The steady-state PL spectra (Fig. S6a) show similar profiles for all HCP-SOs, with two emission bands centred at 465 nm (2.7 eV) and 600 nm (2.1 eV). The intensity ratio *I*_600_/*I*_465_ correlates with the linker : monomer ratio, decreasing from 5.6 for HCP-SO-0.5 to 1.1 for HCP-SO-3.0, suggesting that the two bands originate from the monomer and linker, respectively.

We further determined intensity-averaged lifetimes by analysing decays at 600 nm and 465 nm (Fig. S6b and Table S7). The emission at 465 nm shows shorter lifetimes (<1 ns) than the 600 nm band (2–6 ns), with lifetime ratios of 4–8 for all samples except HCP-SO-3.0. These differences support the assignment of the two emissions to distinct structural units and indicate incomplete electronic communication between linker and monomer, leading to localised excitation and relaxation pathways.

### Photocatalytic hydrogen evolution experiments

**Fig. 2:**
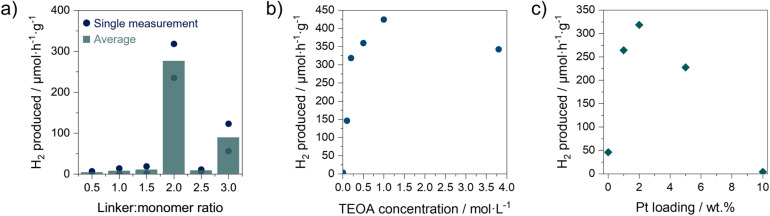
Hydrogen evolution rates of (a) dibenzo[*b*,*d*]thiophene sulfone-based HCPs with varied monomer/linker ratios, (b) HCP-SO-2.0 tested with varied TEOA concentrations, and (c) HCP-SO-2.0 with varied Pt loadings.

The photocatalytic performance of HCP-SOs towards sacrificial hydrogen evolution from water was determined in a 1 : 1 (v/v) mixture of water and methanol, containing triethanolamine (TEOA) at a concentration of 0.2 M, using a custom-built glass reactor and a narrow-band LED as the light source (Fig. S8). Here, triethanolamine acts as the sacrificial reagent that is oxidised instead of water, while methanol assists with the dispersion of the material in water as well as facilitating swelling of the material (Fig. S9), making more of the interface available for proton reduction to occur. In a typical experiment, 2 wt% platinum from H_2_PtCl_6_ was added as co-catalyst by *in situ* photodeposition (see Section 3 of the SI for details). All networks were synthesised in duplicate and their average hydrogen evolution rates after 1 h of irradiation for each sample are shown in Fig. 2a, as well as Table S8.

Despite the narrow band gaps of the HCP-SOs and their absorption extending into the visible light range, no hydrogen evolution was observed under broad-band visible-light irradiation (400–800 nm), whereas illumination at 365 nm enabled measurable activity, indicating more efficient generation of charge carriers under higher-energy excitation conditions. Photocatalytic data presented in Fig. 2 summarises hydrogen evolution rates of the HCP-SOs obtained under 365 nm LED light. HCP-SO-0.5, HCP-SO-1.0, HCP-SO-1.5, and HCP-SO-2.5 displayed only minor photocatalytic activity towards hydrogen evolution, reaching rates of <20 µmol h^−1^ g^−1^. By contrast, average hydrogen evolution rates of 90 µmol h^−1^ g^−1^ were measured for HCP-SO-3.0, whereas best-performing HCP-SO-2.0 reached outstanding 277 µmol h^−1^ g^−1^. This set of results demonstrates that varying the linker/monomer ratio in our HCP-SOs has a pronounced effect on the hydrogen evolution rates. To further elucidate this behaviour, we evaluated additional property–performance correlations for H_2_ production, including BET surface area, pore volumes, sulfur content, particle size, optical band gaps, and *I*_600_/*I*_465_ emission ratios (Fig. S10). Among these descriptors, higher ultramicroporosity and smaller particle size appeared most strongly associated with enhanced H_2_ generation: HCP-SO-2.0, the most active catalyst, exhibited both the highest ultramicropore volume and the smallest particle size, followed by HCP-SO-3.0, which showed with the second-highest activity. This suggests that improved accessibility of active sites, together with reduced diffusion limitations in smaller particles with a higher fraction of ultramicropores, may be responsible for the enhanced photocatalytic performance.

The direct comparison of photocatalytic performance data is near impossible given the variation in experimental conditions as well as experimental set-ups.^52^ In order to benchmark the results obtained here, we determined the performance of TiO_2_ (anatase) under our standard conditions, leading to hydrogen evolution rates of 13.9 mmol h^−1^ g^−1^, still significantly higher than the HCPs herein.

The best performing photocatalyst (HCP-SO-2.0) was further optimised by varying the ratio of both the sacrificial hole scavenger TEOA (Fig. 2b and Table S9) and the Pt co-catalyst (Fig. 2c and Table S10). Increasing TEOA led to improved hydrogen evolution rates until reaching a threshold of 424 µmol h^−1^ g^−1^ for 1.0 M TEOA, revealing the importance of the sacrificial hole scavenger in the system. Similarly, increasing the amount of Pt led to increased hydrogen evolution rates up to a concentration of 2 wt%. Higher co-catalyst concentrations were found to be detrimental to the photocatalytic performance, which can potentially be explained by poorer light absorption upon higher Pt loadings, which has also been previously observed for carbon nitrides.^53^

Control experiments showed no hydrogen was generated in the absence of light. Aqueous methanol solutions and pure water (containing HCP-SO-2.0 but without TEOA and Pt) led to low but measurable hydrogen evolution rates of 30 µmol h^−1^ g^−1^ and 5 µmol h^−1^ g^−1^, respectively. This is rationalised by methanol's ability to act as a hole scavenger, albeit with poorer ability to quench excitons in dibenzo[*b*,*d*]thiophene sulfone materials.^54^

Inspired by these results, we varied the rigidity of the networks and the chemical functionality of HCPs by replacing dibenzo[*b*,*d*]thiophene sulfone with either fluorene, carbazole, dibenzofuran, or dibenzothiophene (experimental details and full polymer characterisation are provided in Section 4 of the SI).^55^ Varying the building blocks of the HCPs led to materials with no significant photocatalytic activity. Interestingly, the introduction of more flexibility into the backbone led to a reduction in the hydrogen evolution rate from an average of 249 ± 41 µmol h^−1^ g^−1^ for the dibenzo[*b*,*d*]thiophene sulfone-based material to just 68 µmol h^−1^ g^−1^ for diphenyl sulfone, confirming the role of the conjugated dibenzo[*b*,*d*]thiophene sulfone moiety for efficient hydrogen production.^56^ As an additional control experiment, we investigated the photocatalytic activity of a non-functionalised hypercrosslinked polymer, synthesised *via* the self-condensation of 4,4′-bis(chloromethyl)-1,1′-biphenyl, which gave hydrogen evolution rates of only 2 µmol h^−1^ g^−1^, further underlining the importance of the dibenzo[*b*,*d*]thiophene sulfone moieties. Although HCPs allow for broad chemical exploration, sulfone groups remained the most active functionality due to their inherent hydrophilicity^57^ and ability to act as an electron transfer site.^56^

**Fig. 3:**
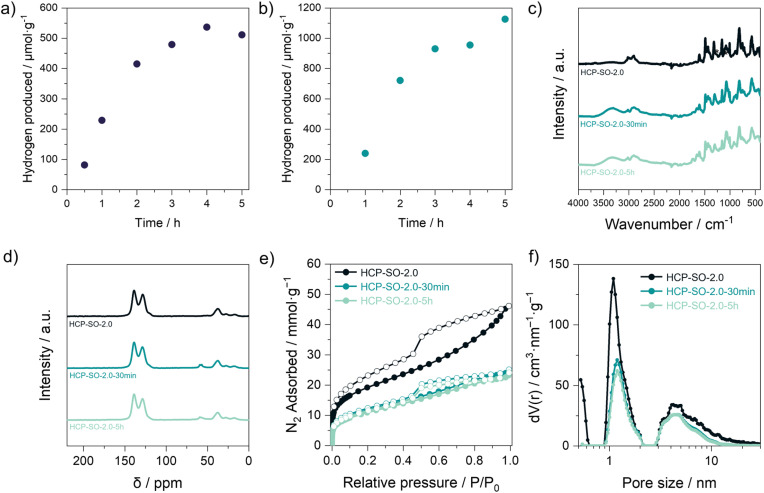
(a) Cumulative amount of hydrogen produced in a long-term experiment using HCP-SO-2.0 as photocatalyst. (b) Cumulative amount of hydrogen produced in a long-term experiment using HCP-SO-2.0 as photocatalyst and nickel(ii) dibutyldithiocarbamate as a singlet oxygen and superoxide anion scavenger. (c) FTIR spectra, (d) ^13^C CP/MAS ssNMR spectra, (e) N_2_ sorption isotherms measured at −196 °C, and (f) QSDFT pore size distribution of HCP-SO-2.0 as synthesised (black), after 30 min of UV irradiation (light blue) and 5 h of UV irradiation (light green).

We explored the stability of sulfone-containing HCPs by employing HCP-SO-2.0 in a hydrogen evolution experiment over 5 h (Fig. 3a and Table S16). During the first two hours of irradiation with 365 nm light, no significant decrease in hydrogen evolution rate was observed. Extending the irradiation time resulted in an appreciable deactivation of the photocatalyst leading to almost complete deactivation after 5 h. To further examine this behaviour, we performed an experiment in which the catalyst was removed from the reaction mixture after 2 h and redispersed in a fresh reaction suspension (Fig. S14). Comparable inactivation was observed after 5 h, suggesting that the loss in activity is intrinsic to the catalyst. Aitchison *et al.* observed a similar deactivation process in dibenzo[*b*,*d*]thiophene sulfone, which they assigned to the oxidation of the active sulfone moiety to [1,1′-biphenyl]-2-sulfonic acid by either singlet oxygen or superoxide anions generated from residual oxygen in the system.^15^ To test whether a similar mechanism was responsible for the deactivation of our photocatalytic system, nickel(ii) dibutyldithiocarbamate was added as a singlet oxygen and superoxide anion scavenger.^58^ The addition of the Ni(ii) species led to a slight increase in the hydrogen evolution rate to 275 ± 58 µmol h^−1^ g^−1^, yet remained within the standard deviation of the measurements relative to the pristine system, while stability was extended to at least 5 h (Fig. 3b). To gain a better understanding of our material after photocatalysis, we analysed HCP-SO-2.0 after 30 min and 5 h of exposure to UV light in the absence of Ni(ii) using FT-IR, ssNMR, TGA, and N_2_ gas sorption. FT-IR (Fig. 3c) and ssNMR (Fig. 3d) analysis showed no significant changes, except for the appearance of additional signals at 3500–3200 cm^−1^ and 59 ppm, respectively, corresponding to OH-groups potentially due to sample decomposition or the adsorption of residual TEOA and/or methanol. Post-catalysis, N_2_ sorption isotherms of HCP-SO-2.0 (Fig. 3e) demonstrated a significant reduction in porosity when compared to the pristine network, resulting in a reduction in BET surface area from 1522 m^2^ g^−1^ to 808 m^2^ g^−1^ and 772 m^2^ g^−1^ after 30 min and 5 h, respectively, suggesting partial pore collapse or blocking (Table S17). Similarly, QSDFT analysis (Fig. 3f) revealed a decrease in both micro- and mesoporosity, which was particularly visible in the range below 1 nm. Thermogravimetric analysis (Fig. S15) revealed a decrease in thermal stability with the degradation onset temperature shifted from >300 °C to only ∼200 °C.

Taken together, the data suggests that the exposure of the polymers to photocatalytic conditions, the basic TEOA, and redox-active radicals generated in the solution may lead to their gradual structural and chemical collapse, which can be somewhat stabilised by the addition of a singlet oxygen and superoxide anion scavenger.

## Conclusions

In this study, we utilised swellable hypercrosslinked polymers as photocatalysts for sacrificial hydrogen evolution from water. A key advantage of this materials platform is its synthetic simplicity and scalability. The networks are accessible *via* a one-step Friedel–Crafts route from cheap, commercially available monomers, avoiding noble metal catalysts or highly functionalised monomers. Using the dibenzo[*b*,*d*]thiophene sulfone motif in a 1 : 2 ratio with 4,4′-bis(chloromethyl)-1,1′-biphenyl gave the material with the highest activity. The catalyst swellability provided interfacial access in solution without relying on nanostructuring strategies. These results also show that measurable HER activity can be achieved in an amorphous network without long-range conjugation. Longer-term experiments showed that the material was unstable under photocatalytic conditions, which could be overcome by the addition of a Ni(ii) complex acting as singlet oxygen or superoxide anion scavenger to prolong catalyst lifetimes. Although the activity of the materials is found to be lower than other leading materials, it showcases the potential of this inexpensive and readily available material class for photocatalytic hydrogen evolution from water.

## Conflicts of interest

The authors declare no competing interests.

## Supplementary Material

TA-014-D6TA01673A-s001

## Data Availability

The data supporting this article have been included as part of the supplementary information (SI). Supplementary information: experimental procedures, synthesis protocols, additional characterisation data (FT-IR, solid-state NMR, XPS, elemental analysis, gas sorption, PXRD, UV-vis spectroscopy), photocatalytic testing details, optimisation studies, and stability experiments (Tables S1–S19 and Fig. S1–S15). See DOI: https://doi.org/10.1039/d6ta01673a.
