# Supplementary material for: Sulfone-decorated hypercrosslinked polymers for sacrificial light-driven hydrogen evolution from water
Source: J Mater Chem A Mater. 2026 Jun 26;14(46):31624–31. doi: 10.1039/d6ta01673a (PMC13325461; doi:10.1039/d6ta01673a)
Supplement: TA-014-D6TA01673A-s001 [file TA-014-D6TA01673A-s001.pdf]

Supplementary Information (SI) for Journal of Materials Chemistry A.
This journal is © The Royal Society of Chemistry 2026

Sulfone-decorated hypercrosslinked polymers for sacrificial light-driven hydrogen evolution from water

Paul Schweng,[a,b] Dominik Eder,[c] Reiner Sebastian Sprick,[d,*] Alexey Cherevan,[c,*] Robert T. Woodward[a,*]

[a] Institute of Materials Chemistry and Research, Faculty of Chemistry, University of Vienna, Währinger Straße 42, 1090, Vienna, Austria.

[b] Vienna Doctoral School in Chemistry, University of Vienna, Währinger Straße 42, 1090, Vienna, Austria.

[c] Institute of Materials Chemistry, TU Wien, Getreidemarkt 9/BC, 1060, Vienna, Austria.

[d] Department of Pure and Applied Chemistry, University of Strathclyde, Glasgow G1 1XL, Scotland, United Kingdom.

Section 1

Materials and Characterisation

Materials

All chemicals were obtained from commercial sources and used without further purification. Dibenzo[*b*,*d*]thiophenone sulfone (97%), diphenyl sulfone (97%), fluorene (98%), carbazole (for synthesis), dibenzofuran (for synthesis), dibenzo[*b*,*d*]thiophene (98%), 4,4‘-bis(chloromethyl)-1,1‘-biphenyl (95%), $FeCl_3$ (reagent grade, 97%), 1,2-dichloroethane (ACS reagent, ≥99%) and chloroplatinic acid solution (8 wt.% in H_2O) were purchased from Sigma-Aldrich. Methanol (≥99.8%) was purchased from Thermo Fisher Scientific. Nickel (II) dibutyldithiocarbamate (≥97%) was sourced from TCI Chemicals. Triethanolamine (≥98%) was purchased from Alfa Aesar.

Characterisation

Fourier-Transform Infrared spectroscopy (FTIR) spectra were acquired at ambient temperature using a Tensor II FTIR spectrometer (Bruker) equipped with a Bruker Optics Platinum ATR module. The measurements were performed in the range of 400 - 4000 cm^{-1} with a spectral resolution of 4 cm^{-1} in a double-sided forward–backward acquisition mode. Each spectrum was averaged over a total of 32 scans using a Blackman-Harris 3-term apodisation function and a zero-filling factor of 4. During each measurement, the instrument was purged with dry air and spectra were recorded and analysed using OPUS 7.5 software.

Solid-state NMR was carried out on a Bruker Avance NEO 500 wide bore system (Bruker BioSpin, Rheinstetten, Germany) using a 4 mm triple resonance magic angle spinning probe. Between 15 - 25 mg of material was packed into a 4 mm zirconia CRAMPS rotor. The resonance frequency for ^{13}C NMR was 125.78 MHz, the MAS rotor spinning was set to 14 kHz. Cross polarisation was achieved by a ramped contact pulse with a contact time of 3 ms. During acquisition ^{1}H was high

power decoupled using SPINAL with 64 phase permutations. The 1H π/2 pulse was 2.5 μs, the relaxation delay was set to 4 s, and with roughly 2000 scans a sufficient signal to noise was achieved. The chemical shifts for ^{13}C are reported in ppm and are referenced external to adamantane by setting the low field signal to 38.48 ppm.

X-Ray photoelectron spectroscopy (XPS) was performed on a Nexsa Photoelectron Spectrometer (Thermo Scientific). High-resolution spectra of carbon (C 1s 279–298 eV), nitrogen (N 1s 392–410 eV), oxygen (O 1s 525–545 eV), sulfur (S 2p 157–175 eV), chlorine (Cl 2p 190-210 eV), and Platinum (Pt 4f 64–86 eV) were recorded with a resolution of 0.1 eV and a pass energy of 50 eV. All measurements were performed using Al-Kα X-rays with a spot size of 400 μm. Spectra were analysed using Avantage S4 software (v5.9931, Thermo Fisher Scientific) and the atomic composition of the sample was determined from the peak area using the integrated scaling factor database ALTHERMO1.

Elemental analysis was performed using a Eurovector EA 3000 CHNS-O Elemental Analyser. Between 0.75 and 3.0 mg of each sample was weighed into tin vials (4×6 mm). Samples were weighed using a micro balance (Sartorius, ME 5 OCE). The operating temperatures for the combustion and reduction were 1000 °C (1480 °C for O analysis) and 750 °C, respectively, with high purity helium (99.999+) used as a carrier gas. All samples were measured at least in triplicate.

The nitrogen adsorption–desorption isotherms were measured at −196 °C using a 3Flex (Micromeritics), or a TriStar II (Micromeritics) for sample repeats. Before the measurement, 50 to 100 mg of sample was degassed at 120 °C under N_2 flow for 14 h using a FlowPrep 060 (Micromeritics). The networks' specific surface areas (SSA) were determined *via* the Brunauer–Emmett–Teller (BET) method in the relative pressure P/P0 range 0.05 to 0.3. Pore size distributions (PSDs) were determined from the N_2 adsorption branch of the isotherm using the quenched solid density functional theory (QSDFT) model. The total pore volume VP, micropore

volume VMic, and mesopore volume VMes were determined from the cumulative pore volumes calculated by QSDFT model in the relevant pore width ranges. Thermogravimetric analysis was carried out on a Discovery TGA (TA instrumentation). Approximately 10 mg of each sample was heated at a rate of 10 °C min^{-1} from room temperature to 800 °C under nitrogen gas flow (100 mL min^{-1}) and the change in weight was monitored gravimetrically.

Powder X-ray diffraction (PXRD) measurements were conducted in reflection (Bragg-Brentano HD) geometry on a PANalytical EMPYREAN diffractometer equipped with a PIXcel3D detector (Malvern, PANalytical). The measurements were recorded from 2° - 50° 2Θ in continuous measurement mode, a step size of 0.0131°, and a data time per step of 720 s using Cu Kα1+2 radiation, which was generated in an X-ray tube operated at 45 kV and 40 mA.

UV–Vis diffuse reflectance spectra were measured using a V670 UV–vis spectrometer (Jasco) with a diffuse reflectance unit containing an integrating sphere. $MgSO_4$ was used as a white reference material, The wavelength of the incident light ranged from 250–800 nm and the measured reflectance was converted using the Kubelka–Munk function.

Both steady-state and time-resolved measurements were conducted on the powderous samples using a FluoTime 300 spectrometer. For steady-state measurements, a 300 W coaxial UV-Xenon arc lamp with a high-resolution double monochromator was used as light source and 365 nm was chosen as the excitation wavelength following the setup of our photocatalytic measurements. Time-resolved measurements were performed by means of time-correlated single photon counting (TCSPC) using a PDL 820 laser driver with an LDH-P-C-375 laser head with the actual emission maximum centred at 377.8 nm. Emitted photons were detected with a PMA Hybrid 07 detector, equipped with a high-resolution double monochromator.

Section 2

Sulfone containing hypercrosslinked polymer

Synthesis of sulfone-containing hypercrosslinked polymers with varying ratios

Dibenzo[*b*,*d*]thiophenone sulfone (0.216 g, 1 mmol) and 4,4′-bis(chloromethyl)-1,1′-biphenyl (0.5, 1.0, 1.5, 2.0, 2.5, 3.0 mmol) were dissolved in 1,2-dichloroethane (4 mL) at ambient temperature. Upon dissolution, $FeCl_3$ (0.5, 1.0, 1.5, 2.0, 2.5, 3.0 mmol) was quickly added and left stirring for a further 15 min at room temperature before heating at 80 °C overnight. The resulting black material was filtered off and washed multiple times with methanol. Following this, the material was transferred to a cellulose thimble and a Soxhlet extraction with methanol was performed for 24 h. The polymer was then dried for a few hours at room temperature before drying under vacuum at 80 °C overnight. The dry material was then lightly ground using a pestle and mortar, yielding the final products as brown powders (Yields in Table S1)

Table S1. Yields of HCPs based on dibenzo[*b*,*d*]thiophene sulfone with varied monomer:linker ratios

	monomer:linker ratio	Yield (g)	Yield (%)
HCP-SO-0.5	1:0.5	0.070	23
HCP-SO-1.0	1:1.0	0.138	35
HCP-SO-1.5	1:1.5	0.249	51
HCP-SO-2.0	1:2.0	0.367	64
HCP-SO-2.5	1:2.5	0.496	75
HCP-SO-3.0	1:3.0	0.575	77

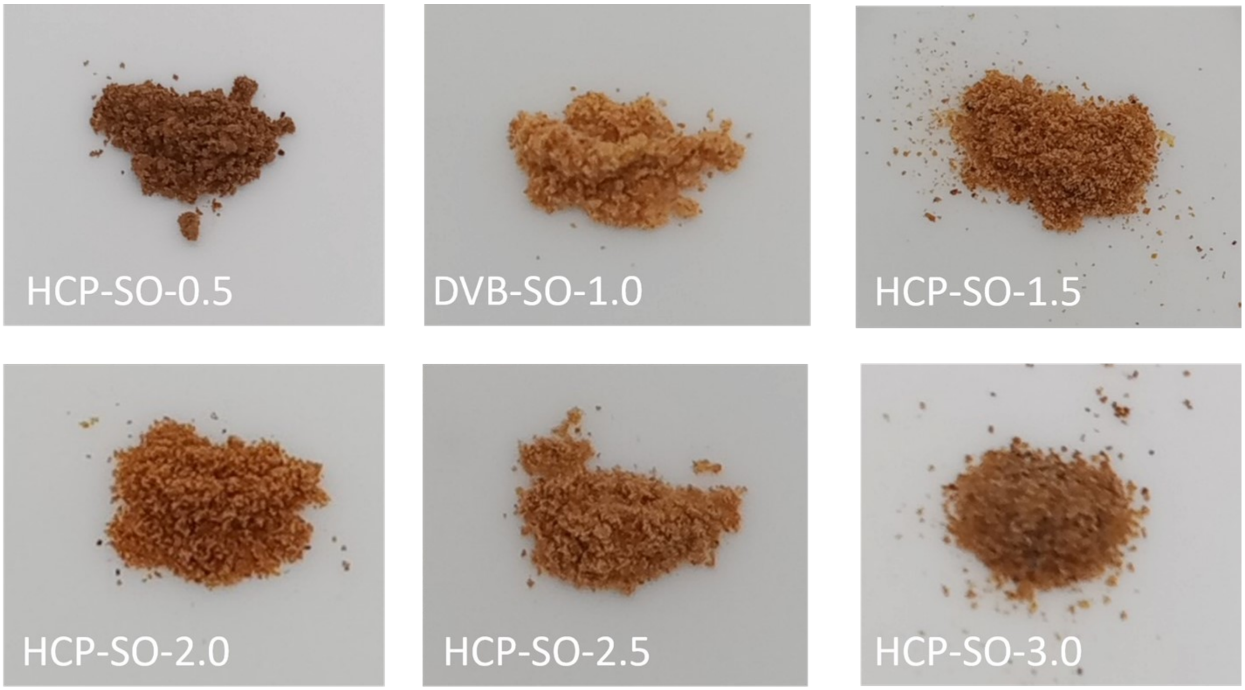

Figure S1. Photographs of HCPs based on dibenzo[*b*,*d*]thiophene sulfone with varied monomer/linker ratios.

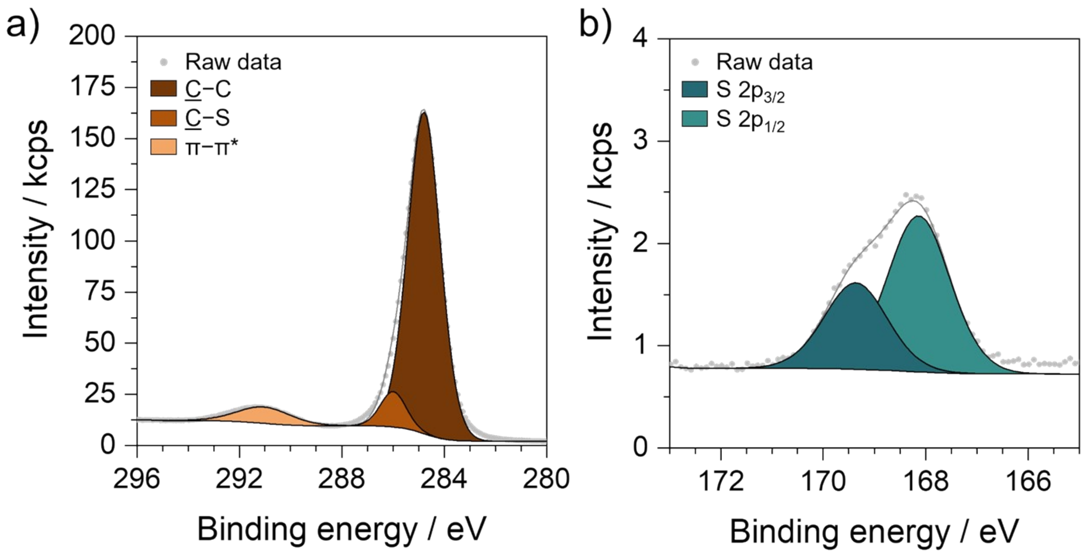

Figure S2. Representative X-ray photoelectron spectra of a) high-resolution C 1s spectrum of HCP-SO-2.0, and b) high-resolution S 2p spectrum of HCP-SO-2.0. (grey dots represent raw data).

Table S2. Surface elemental composition of HCPs based on dibenzo[*b*,*d*]thiophene sulfone with varying monomer/linker ratios determined by XPS.

	C (wt.%)	N (wt.%)	O (wt.%)	S (wt.%)	Cl (wt.%)
HCP-SO-0.5	92.83 ± 0.26	0.09 ± 0.01	3.49 ± 0.04	2.09 ± 0.09	1.38 ± 0.14
HCP-SO-1.0	93.83 ± 0.27	0.08 ± 0.02	2.81 ± 0.13	1.92 ± 0.05	1.37 ± 0.11
HCP-SO-1.5	92.94 ± 0.14	0.10 ± 0.02	3.22 ± 0.10	1.66 ± 0.08	2.14 ± 0.06
HCP-SO-2.0	92.21 ± 0.59	0.09 ± 0.02	3.42 ± 0.16	1.58 ± 0.13	2.67 ± 0.25
HCP-SO-2.5	92.51 ± 0.33	0.10 ± 0.04	3.51 ± 0.22	1.29 ± 0.01	2.61 ± 0.15
HCP-SO-3.0	91.29 ± 0.01	0.15 ± 0.03	3.95 ± 0.01	1.16 ± 0.06	3.47 ± 0.02

Table S3. Bulk elemental composition of HCPs based on dibenzo[*b*,*d*]thiophene sulfone with varying monomer/linker ratios determined by EA.

	C (wt.%)	N (wt.%)	O (wt.%)	S (wt.%)	H (wt.%)
HCP-SO-0.5	87.78 ± 0.47	< 0.05	3.05 ± 0.41	1.87 ± 0.07	5.08 ± 0.05
HCP-SO-1.0	88.69 ± 0.50	< 0.05	2.55 ± 0.14	1.61 ± 0.04	5.17 ± 0.09
HCP-SO-1.5	88.65 ± 0.20	< 0.05	2.75 ± 0.18	1.56 ± 0.03	5.14 ± 0.05
HCP-SO-2.0	87.98 ± 0.52	< 0.05	2.75 ± 0.13	1.48 ± 0.06	5.13 ± 0.04
HCP-SO-2.5	87.93 ± 0.21	< 0.05	2.44 ± 0.13	1.01 ± 0.02	5.15 ± 0.04
HCP-SO-3.0	89.25 ± 0.05	< 0.05	2.25 ± 0.41	0.98 ± 0.03	5.33 ± 0.01

Table S4. Theoretical elemental composition of HCPs assuming complete conversion

	C (wt.%)	N (wt.%)	O (wt.%)	S (wt.%)	H (wt.%)
HCP-SO-0.5	74.48	-	10.45	10.46	4.61
HCP-SO-1.0	78.76	-	8.07	8.09	5.08
HCP-SO-1.5	81.45	-	6.58	6.59	5.39
HCP-SO-2.0	83.30	-	5.55	5.56	5.59
HCP-SO-2.5	84.65	-	4.80	4.81	5.74
HCP-SO-3.0	85.68	-	4.23	4.24	5.86

Table S5. Summary of nominal and experimental linker:monomer ratios, monomer incorporation percentage and crosslinking density in HCP-SO-Xs. The crosslinking density is defined as the number of crosslinks per aromatic ring. All experimental values were calculated using elemental compositions determined from XPS.

	Linker:monomer ratio		Monomer incorporation (%)	Crosslinking density	
	Nominal	Experimental		Nominal	Experimental
HCP-SO-0.5	0.5	7.7	17	0.33	0.85
HCP-SO-1.0	1.0	8.5	21	0.50	0.86
HCP-SO-1.5	1.5	9.9	23	0.60	0.86
HCP-SO-2.0	2.0	10.9	25	0.67	0.85
HCP-SO-2.5	2.5	13.0	25	0.71	0.86
HCP-SO-3.0	3.0	14.1	27	0.75	0.84

Table S6. Summary of textural properties of HCPs with varying monomer/linker ratios. Data includes BET specific surface area, SSA_{BET}, volume of micropores, V_{MICRO}, and total pore volume, V_{TOT}.

	SSA_{BET} ($m^2\ g^{-1}$)	V_{MICRO} ($cm^3\ g^{-1}$)	V_{TOT} ($cm^3\ g^{-1}$)
HCP-SO-0.5	1511 ± 49	0.18 ± 0.00	1.51 ± 0.14
HCP-SO-1.0	1563 ± 29	0.18 ± 0.01	1.74 ± 0.09
HCP-SO-1.5	1591 ± 13	0.18 ± 0.00	1.80 ± 0.01
HCP-SO-2.0	1522 ± 137	0.18 ± 0.01	1.64 ± 0.25
HCP-SO-2.5	1681 ± 6	0.20 ± 0.01	1.98 ± 0.14
HCP-SO-3.0	1599 ± 62	0.20 ± 0.01	1.47 ± 0.07

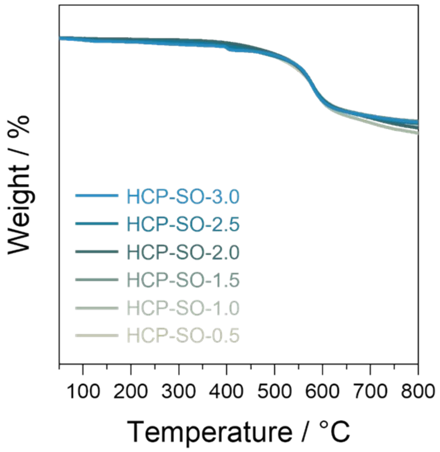

Figure S3. Thermogravimetric analysis of HCP-SOs under nitrogen with a heating rate of 10 °C min^{-1}.

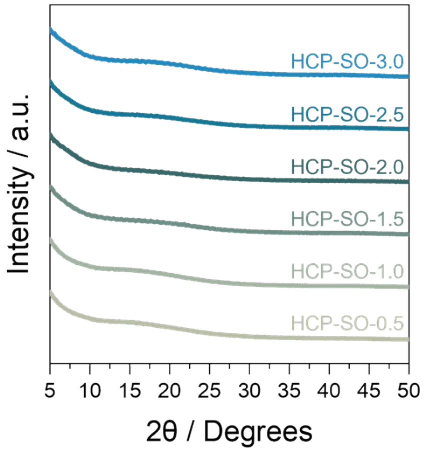

Figure S4. Powder X-ray diffractograms of HCP-SOs.

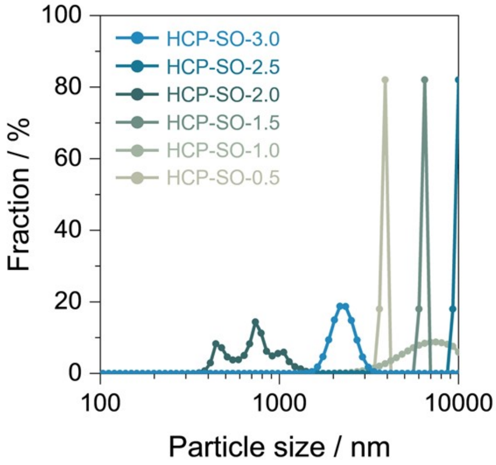

Figure S5. Particle size distribution of HCP-SOs in suspension determined by dynamic light scattering.

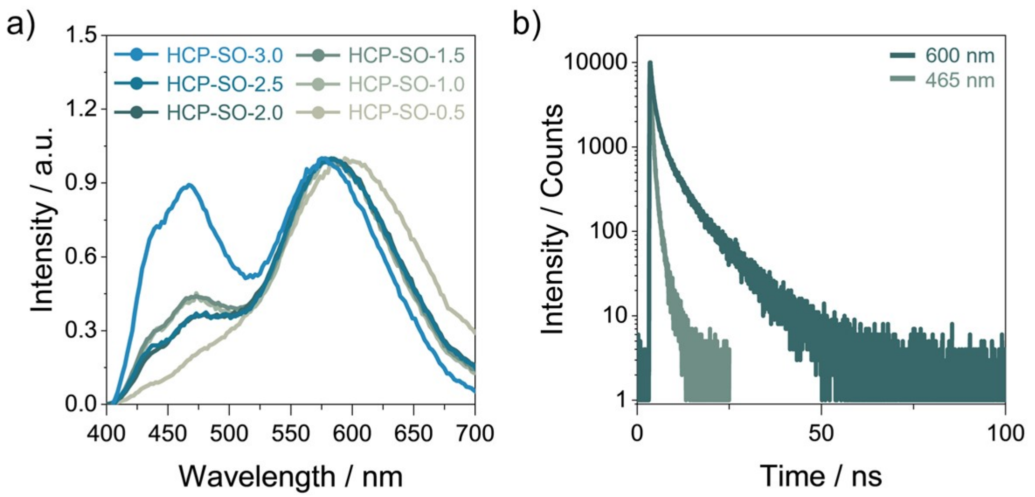

Figure S6. a) Steady-state PL spectra of HCP-SOs and b) representative time-resolved PL emission spectra of HCP-SO-2.0 using either 600 nm or 465 nm excitation.

Table S7. PL data, including the intensity ratio of the 600 nm and 465 nm peaks (I_{600}/I_{465}) as well as time-resolved decays-derived intensity-averaged lifetimes recorded at 465 (LT_{465}) and 600 nm (LT_{600}).

	I_{600}/I_{465}	LT_{465} (ns)	LT_{600} (ns)
HCP-SO-0.5	5.6	0.48	2.02
HCP-SO-1.0	2.3	0.83	6.26
HCP-SO-1.5	2.2	-	5.43
HCP-SO-2.0	2.7	0.77	4.98
HCP-SO-2.5	2.5	0.77	4.44
HCP-SO-3.0	1.1	1.06	1.03

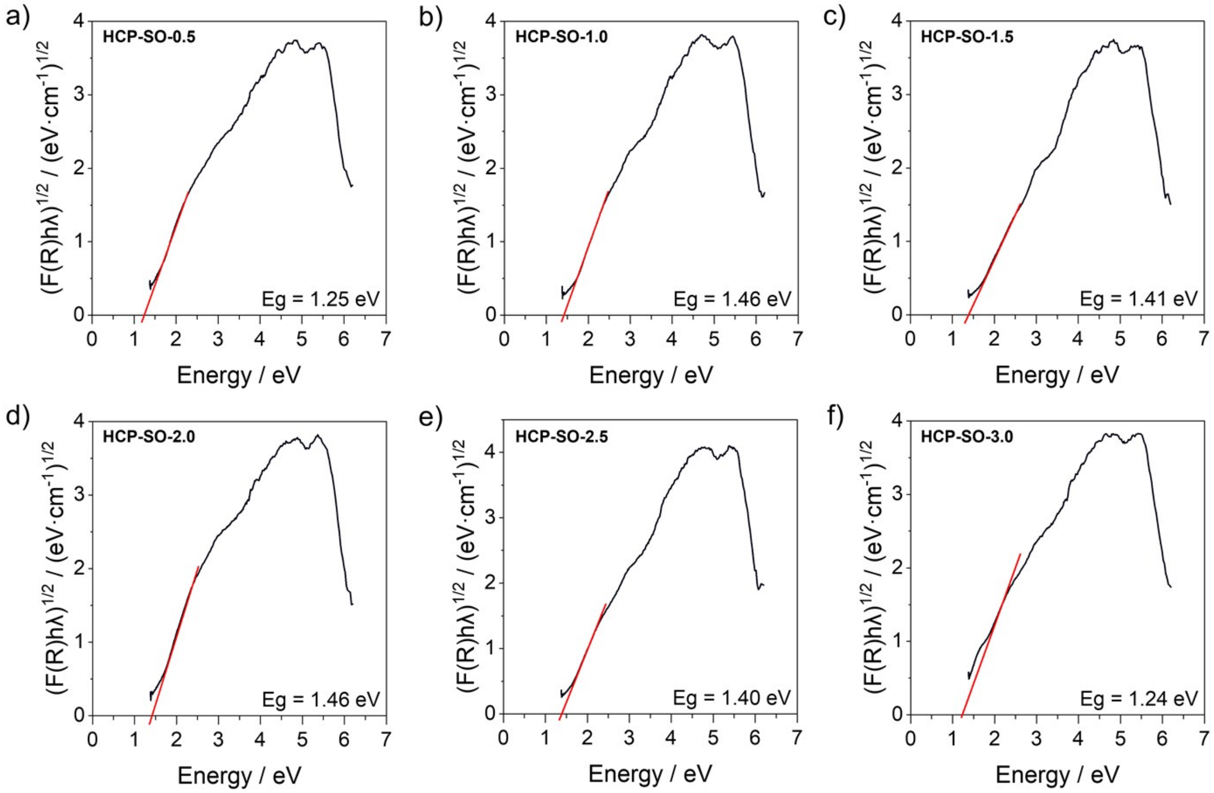

Figure S7. Tauc plots obtained from UV/vis diffuse reflectance spectra of HCP-SOs.

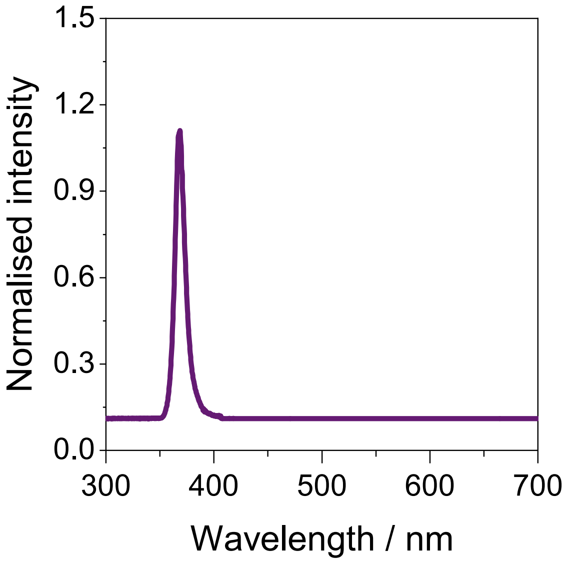

Figure S8. Output spectrum of the narrow-band LED lamp light source (SOLIS-365C, Thorlabs) employed for photocatalytic experiments.

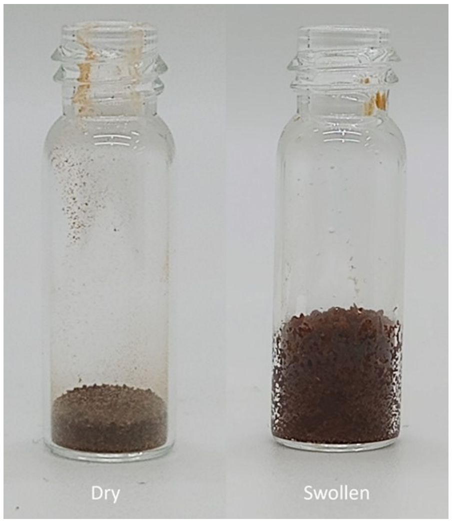

Figure S9. Photographic images of HCP-SO-2.0 after drying at 80 °C under vacuum (left), and after the addition of 0.5 mL of a 1:1 (v/v) methanol–water mixture containing triethanolamine (TEOA) at a concentration of 0.2 M (right).

Section 3

Photocatalytic hydrogen evolution reaction

Photocatalytic hydrogen evolution experiments

Photocatalytic hydrogen evolution reaction experiments were performed using a custom-built glass reactor (9 mL), illuminated from the side with a narrow-band LED centred at 365 nm. The reactor temperature was maintained at 15 °C using a Lauda water-cooling system, and the suspension was stirred at 300 rpm. In a typical experiment, 2 mg of photocatalyst was dispersed by ultrasonication for 3 min in 2 mL of a 1:1 (v/v) methanol–water mixture containing triethanolamine (TEOA) at a standard concentration of 0.2 M. Subsequently, an aqueous H_2PtCl_6 solution was added to achieve Pt co-catalyst loading *via* photodeposition (standard condition: 2 wt.% Pt). The reactor was sealed and purged with argon (10 mL min^{-1}) for 10 min. Before illumination, a 200 µL headspace sample was taken using a gas-tight Hamilton syringe and analysed by gas chromatography (Shimadzu GC-2030; ShinCarbon Micropacked-ST column; barrier discharge ionization detector, BID) to confirm successful degassing and the absence of hydrogen. The light source was then turned on, and 200 µL headspace samples were periodically collected to monitor photocatalytic hydrogen production over time. Gas chromatography data (ppm H_2) were converted to µmol using the ideal gas law, considering the headspace volume. Hydrogen evolution rates were normalised by photocatalyst mass to enable comparison with literature reports.

Table S8. Summary of the Hydrogen evolution rates obtained for HCP-SOs based on dibenzo[*b*,*d*]thiophene sulfone with varying monomer:linker ratios.

	HER rate (µmol h^{-1} g^{-1})		
	Set 1	Set 2	Average
HCP-SO-0.5	1	7	4
HCP-SO-1.0	14	1	8
HCP-SO-1.5	19	2	11
HCP-SO-2.0	220	278	249
HCP-SO-2.5	11	6	9
HCP-SO-3.0	56	123	90

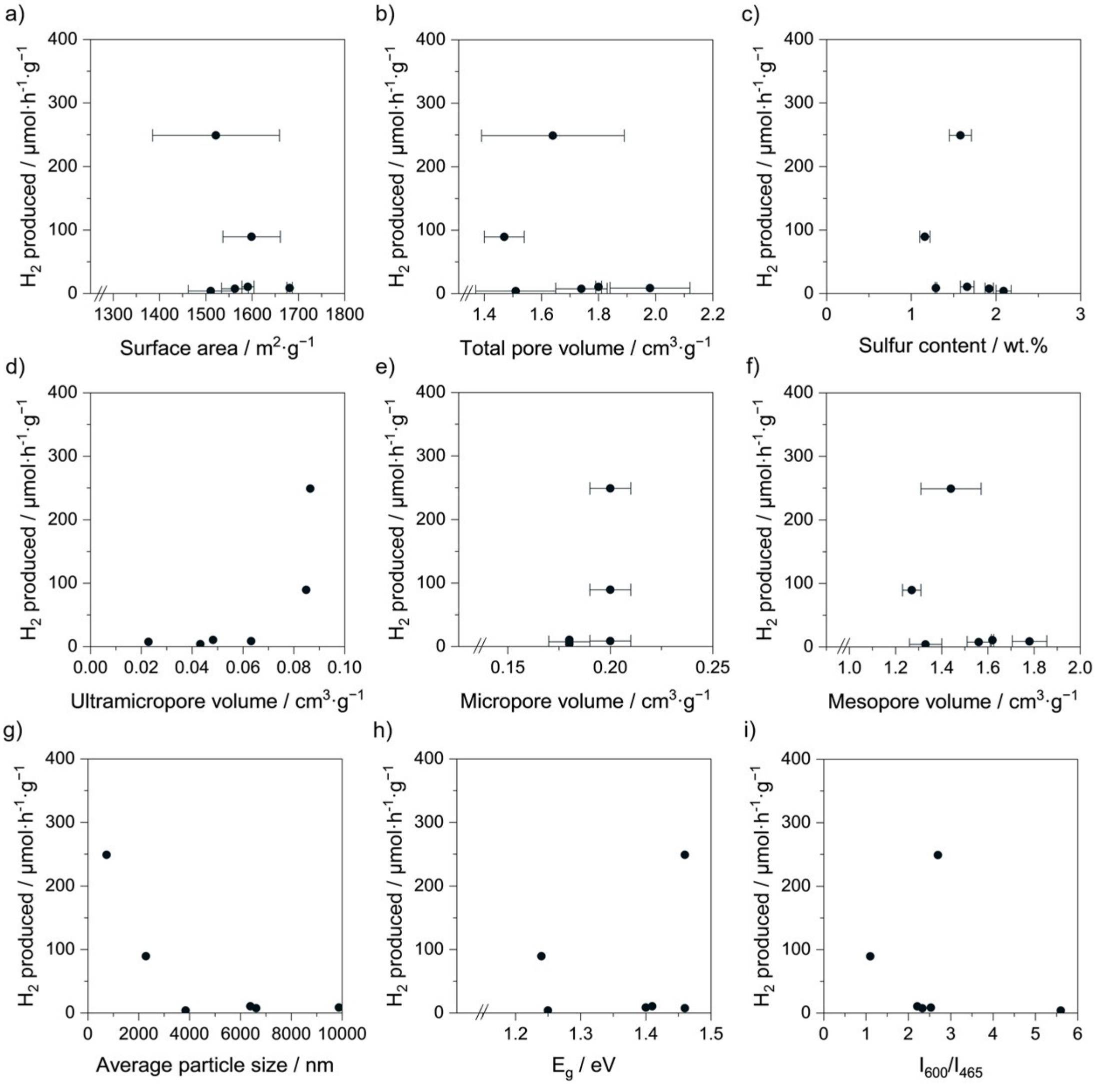

Figure S10. HER activity of HCP-SOs plotted against a) BET surface area, b) total pore volume, c) sulfur content, d) ultramicropore volume, e) micropore volume, f) mesopore volume, g) average particle size determined from DLS, h) DRS band gap E_g, and i) I_{600}/I_{465} emission ratios determined from PL.

Table S9. Hydrogen evolution rate obtained using HCP-SO-2.0 depending on the TEOA concentration

TEOA concentration ($mol\ L^{-1}$)	HER rate ($\mu mol\ h^{-1}\ g^{-1}$)
0.0	3
0.1	146
0.2	318
0.5	360
1.0	424
3.8	342

Table S10. Hydrogen evolution rate obtained using HCP-SO-2.0 depending on the amount of Pt

Amount of Pt (wt.%)	HER rate ($\mu mol\ h^{-1}\ g^{-1}$)
0	46
1	264
2	318
5	227
10	4

Section 4

Variation of the monomeric active site

Synthesis of hypercrosslinked polymers with variation of the monomeric active site

The monomer, either dibenzo[b,d]thiophene sulfone (0.616 g, 2,5 mmol), Diphenyl sulfone (0.546, 2.5 mmol), Fluorene (0.416, 2.5 mmol), Carbazole (0.418, 2.5 mmol), Dibenzofuran (0.421, 2.5 mmol), or Dibenzothiophene (0.461, 2.5 mmol), and the linker, 4,4′-Bis(chloromethyl)-1,1′-biphenyl (1.256 g, 5 mmol) were dissolved in 1,2-dichloroethane (10 mL) at ambient temperature. Upon complete dissolution, $FeCl_3$ (0,811 g, 5 mmol) was quickly added and left stirring for further 15 min at room temperature before heating at 80°C overnight. The resulting black polymer was filtered off and washed multiple times with Methanol until the filtrate didn't appear yellow anymore. The extraction was then continued using a Soxhlet apparatus and Methanol for further 24 h. To remove excess methanol, the polymer was allowed to dry for a few hours at room temperature before it was dried in a vacuum oven at 80 °C overnight. The dry network was then lightly ground using a pestle and mortar, yielding the final product as a fine powder (Yields in Table S11).

Table S11. Yields obtained for HCPs based on various monomers

	Monomer	Yield (g)	Yield (%)
HCP-SO	Dibenzo[*b*,*d*]thiophene sulfone	0.987	69
HCP-SO-f	Diphenyl sulfone	0.810	56
HCP-C	9*H*-Fluorene	1.378	105
HCP-N	9*H*-Carbazole	1.149	88
HCP-O	Dibenzo[*b*,*d*]furan	1.257	96
HCP-S	Dibenzo[*b*,*d*]thiophene	1.319	98

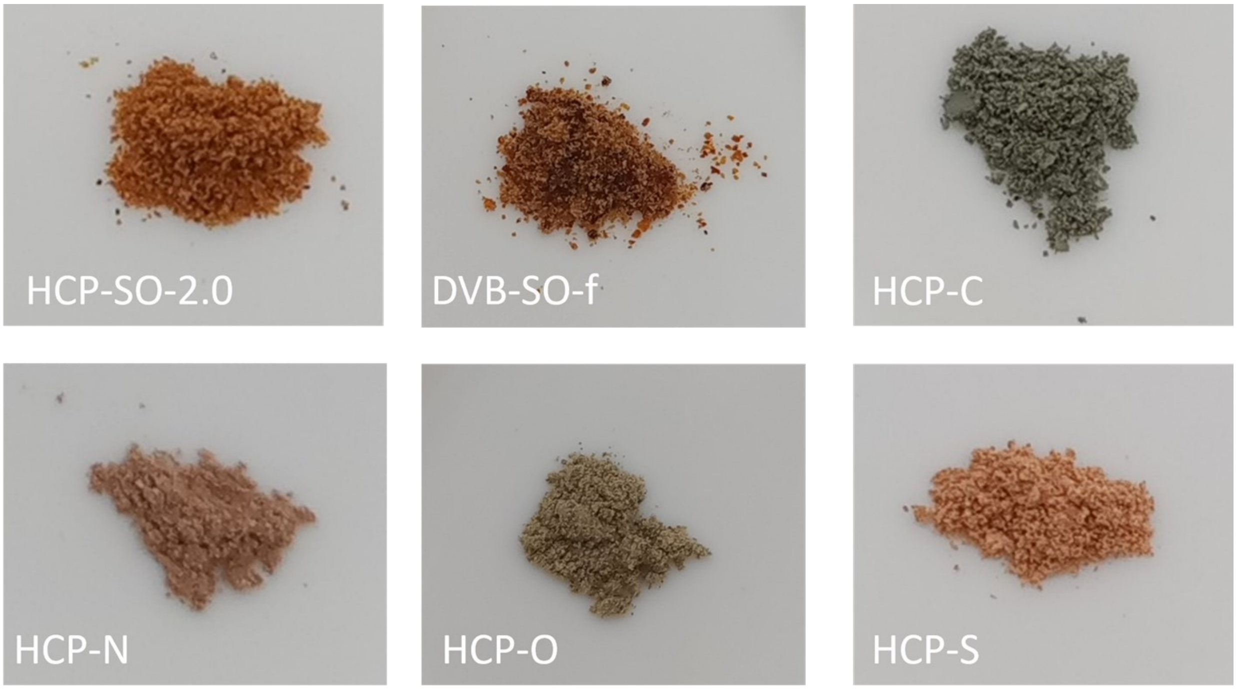

Figure S11. Photographs of HCPs based on various monomers.

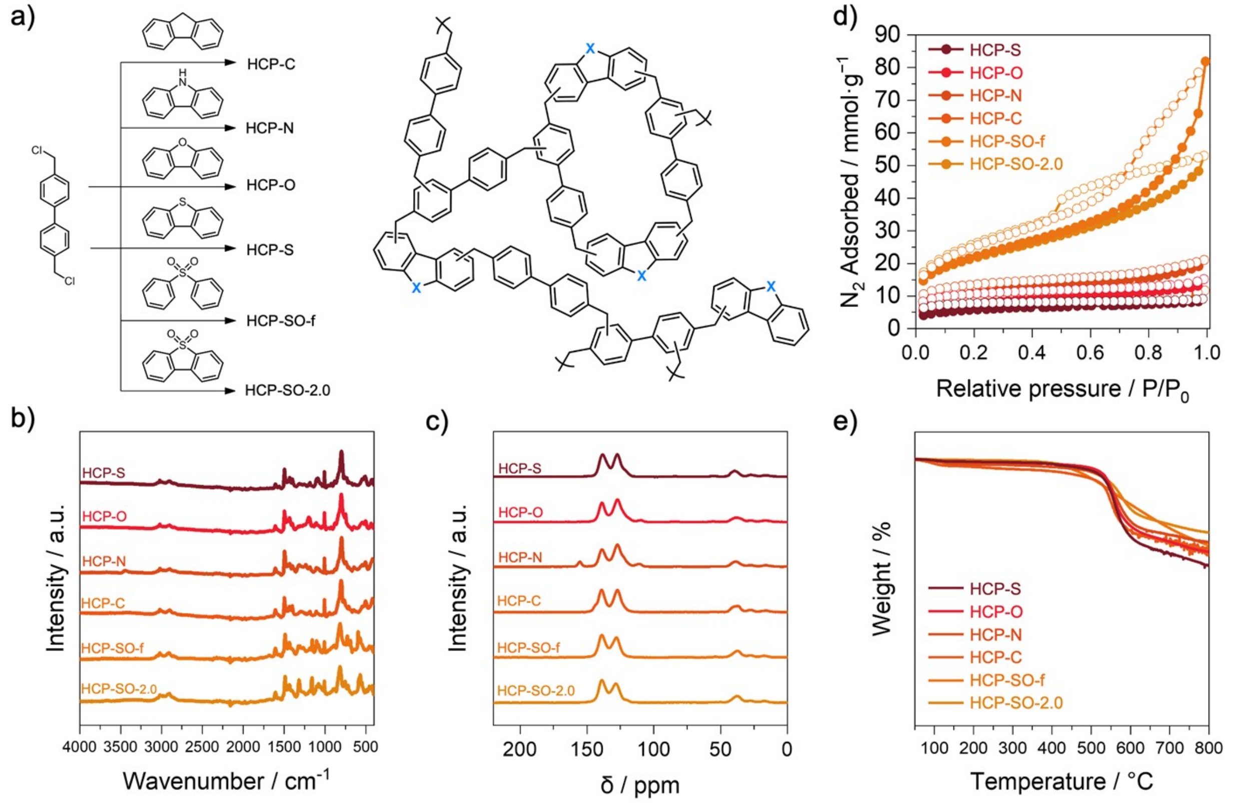

Figure S12. a) General reaction scheme for the synthesis of HCPs based on various monomers. b) FTIR spectra, c) ^{13}C CP/MAS ssNMR spectra, d) N_2 isotherms measured at −196 °C, and e) TGA under N_2 flow of HCP-S, HCP-O, HCP-N, HCP-C, HCP-SO-f, and HCP-SO-2.0

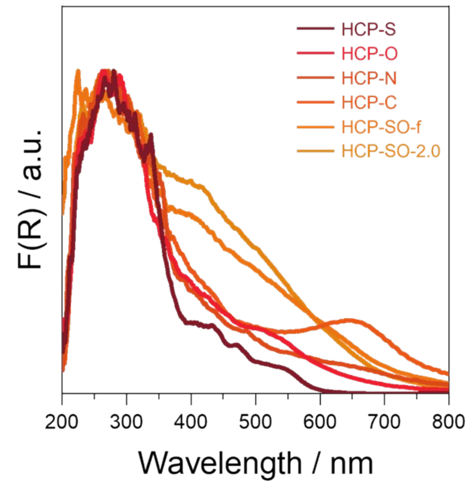

Figure S13. UV/Vis diffuse reflectance spectroscopy of HCP-S, HCP-O, HCP-N, HCP-C, HCP-SO-f, and HCP-SO-2.0

Table S12. Bulk elemental composition of of HCP-S, HCP-O, HCP-N, HCP-C, HCP-SO-f, and HCP-SO-2.0 determined by EA.

	C (wt.%)	H (wt.%)	N (wt.%)	O (wt.%)	S (wt.%)
HCP-SO-2.0	87.98 ± 0.52	5.13 ± 0.04	< 0,05	2.75 ± 0.13	1.48 ± 0.06
HCP-SO-f	88.34 ± 0.40	5.15 ± 0.06	< 0,05	2.31 ± 0.01	0.76 ± 0.02
HCP-C	87.13 ± 0.21	5.25 ± 0.03	< 0,05	2.03 ± 0.27	< 0,02
HCP-N	88.50 ± 0.25	5.26 ± 0.02	2.67 ± 0.02	1.27 ± 0.24	< 0,02
HCP-O	89.02 ± 0.41	5.06 ± 0.06	< 0,05	3.88 ± 0.01	< 0,02
HCP-S	85.58 ± 0.19	4.91 ± 0.02	< 0,05	1.24 ± 0.03	5.58 ± 0.06

Table S13. Surface elemental composition of of HCP-S, HCP-O, HCP-N, HCP-C, HCP-SO-f, and HCP-SO-2.0 determined by XPS.

	C (wt.%)	N (wt.%)	O (wt.%)	S (wt.%)	Cl (wt.%)
HCP-SO-2.0	92.21 ± 0.59	0.09 ± 0.02	3.42 ± 0.16	1.58 ± 0.13	2.67 ± 0.25
HCP-SO-f	93.60 ± 0.16	0.10 ± 0.03	3.10 ± 0.04	0.94 ± 0.05	2.27 ± 0.11
HCP-C	96.23 ± 0.16	0.10 ± 0.01	< 0,05	< 0,05	3.68 ± 0.15
HCP-N	97.23 ± 0.08	1.78 ± 0.04	< 0,05	< 0,05	1.00 ± 0.04
HCP-O	92.90 ± 0.25	0.16 ± 0.05	4.37 ± 0.17	< 0,05	2.56 ± 0.06
HCP-S	95.34 ± 0.21	0.11 ± 0.04	< 0,05	3.50 ± 0.02	1.07 ± 0.27

Table S14. Summary of textural properties of HCP-S, HCP-O, HCP-N, HCP-C, HCP-SO-f, and HCP-SO-2.0. Data includes BET specific surface area, SSA_{BET}, volume of micropores, V_{MICRO}, and total pore volume, V_{TOT}.

	SSA_{BET} ($m^2\ g^{-1}$)	V_{MICRO} ($cm^3\ g^{-1}$)	V_{TOT} ($cm^3\ g^{-1}$)
HCP-SO-2.0	1522 ± 137	0.18 ± 0.01	1.64 ± 0.25
HCP-SO-f	1764	0.18	2.29
HCP-C	547	0.11	0.37
HCP-N	880	0.20	0.66
HCP-O	611	0.11	0.46
HCP-S	852	0.10	0.29

Table S15. Summary of the Hydrogen evolution rates obtained with HCP-S, HCP-O, HCP-N, HCP-C, HCP-SO-f, and HCP-SO-2.0.

	Monomer	HER rate ($\mu mol\ h^{-1}\ g^{-1}$)
HCP-SO-2.0	Dibenzo[*b*,*d*]thiophene sulfone	249 ± 41
HCP-SO-f	Diphenyl sulfone	68
HCP-C	9*H*-Fluorene	11
HCP-N	9*H*-Carbazole	1
HCP-O	Dibenzo[*b*,*d*]furan	2
HCP-S	Dibenzo[*b*,*d*]thiophene	10

Section 5

Longer-term stability experiments

Table S16. Cumulative amount of hydrogen produced from HCP-SO-2.0 over long-term irradiation

Time (h)	Hydrogen produced ($\mu mol\ g^{-1}$)
0	0
0.5	82
1	229
2	415
3	479
4	537
5	511

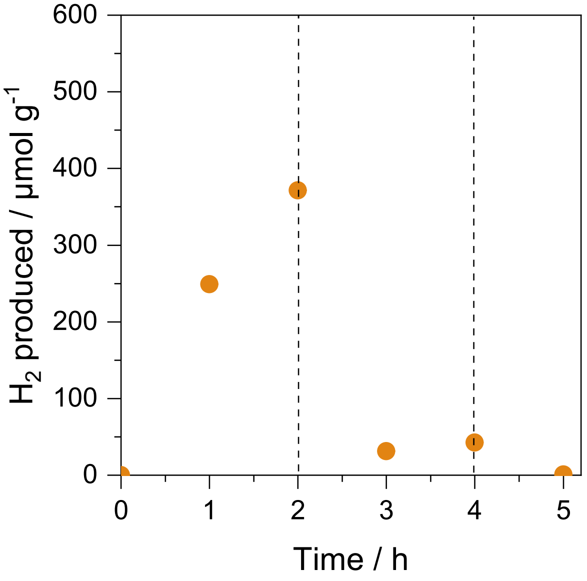

Figure S14. Cumulative hydrogen production in a long-term experiment using HCP-SO-2.0 as the photocatalyst. Dashed lines indicate the removal of the photocatalyst and its subsequent redispersion in a fresh suspension.

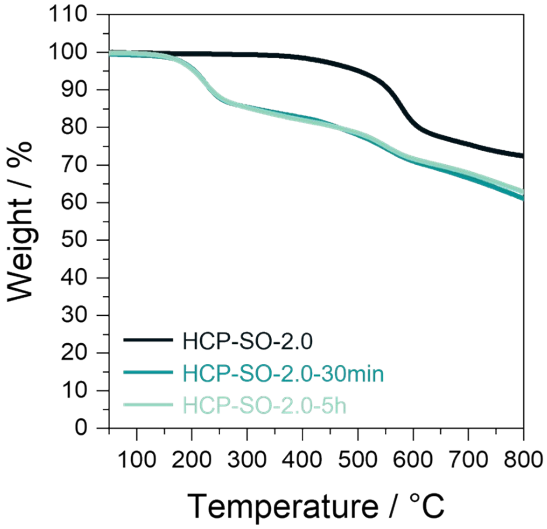

Figure S15. Thermogravimetric curves of HCP-SO-2.0 as synthesised (black), after 30 minutes of UV irradiation (light blue) and 5 hours of UV irradiation (light green).

Table S17. Summary of textural properties of HCP-SO-2.0 as synthesised, after 30 min of UV irradiation and after 5 h of UV irradiation. Data includes BET specific surface area, SSA_{BET}, volume of micropores, V_{MICRO}, and total pore volume, V_{TOT}.

	SSA_{BET} ($m^2\ g^{-1}$)	V_{MICRO} ($cm\ g^{-1}$)	V_{TOT} ($cm^3\ g^{-1}$)
HCP-SO-2.0	1522 ± 137	0.18 ± 0.01	1.64 ± 0.25
HCP-SO-2.0-30min	808	0.05	0.70
HCP-SO-2.0-5h	772	0.05	0.65

Table S18. Bulk elemental composition of HCP-SO-2.0 as synthesised, after 30 min of UV irradiation and after 5 h of UV irradiation as determined by EA

	C (wt. %)	H (wt. %)	N (wt. %)	O (wt. %)	S (wt. %)
HCP-SO-2.0	87.98 ± 0.52	5.13 ± 0.04	< 0,05	2.75 ± 0.13	1.48 ± 0.06
HCP-SO-2.0-30min	80.41 ± 0.13	5.63 ± 0.02	1.62 ± 0.03	7.97 ± 0.81	1.16 ± 0.01
HCP-SO-2.0-5h	80.17 ± 0.25	5.59 ± 0.02	1.78 ± 0.03	18.25 ± 0.74	1.08 ± 0.01

Table S19. Surface elemental composition of HCP-SO-2.0 as synthesised, after 30 min of UV irradiation and after 5 h of UV irradiation as determined by XPS.

	C (wt.%)	N (wt.%)	O (wt.%)	S (wt.%)	Cl (wt.%)	Pt (wt.%)
HCP-SO-2.0	92.21 ± 0.59	0.09 ± 0.02	3.42 ± 0.16	1.58 ± 0.13	2.67 ± 0.25	-
HCP-SO-2.0-30min	87.56 ± 0.11	1.38 ± 0.16	6.84 ± 0.17	1.55 ± 0.04	1.17 ± 0.06	1.15 ± 0.01
HCP-SO-2.0-5h	86.56 ± 0.66	1.58 ± 0.07	8.17 ± 0.32	1.72 ± 0.15	0.66 ± 0.01	0.99 ± 0.01
